# Association of maternal mental health and drinking/smoking with adolescents’ mental health based on the Korea National Health and Nutrition Examination Survey

**DOI:** 10.3389/fpsyt.2023.1087300

**Published:** 2023-06-20

**Authors:** Junghan Lee, Kyungchul Song, Soyoung Jeon, Hye Sun Lee, San Lee, Ho-Seong Kim, Hyun Wook Chae

**Affiliations:** ^1^Department of Psychiatry, Institute of Behavioral Science in Medicine, Yonsei University College of Medicine, Seoul, Republic of Korea; ^2^Department of Pediatrics, Yonsei University College of Medicine, Seoul, Republic of Korea; ^3^Biostatistics Collaboration Unit, Yonsei University College of Medicine, Seoul, Republic of Korea

**Keywords:** socioeconomic factors, adolescents, stress, depression, suicidal ideation

## Abstract

**Introduction:**

Depression is one of the major concerns in adolescence, with a global prevalence of approximately 5%. Diverse environmental factors can affect the development of depression depending on the individual developmental stage.

**Methods:**

Using data from the Korea National Health and Nutrition Examination Survey (KNHANES), we aimed to investigate the association between socioeconomic factors and mental health in a population of non-clinically ill adolescents in Korea totaling 6,261 adolescents aged 12–18 years.

**Results:**

Drinking, smoking, stress, depressed mood, suicidal ideation in adolescents, and stress, depressed mood, and suicidal ideation in mothers were identified as factors associated with adolescent depression. In addition to depressed mood and suicidal ideation, the higher perception of stress in mothers was related to higher stress perception, depressed mood, and suicidal ideation in adolescents. The association of adolescents’ mental health with fathers’ mental health was weaker than that with mothers’ mental health. Additionally, increased smoking and drinking were commonly reported in adolescents with higher stress perception, depressed mood, and suicidal ideation.

**Discussion:**

We conclude that close monitoring of mental health is required for adolescents with drinking and smoking habits and mothers with mental health problems.

## 1. Introduction

Depression is a major psychiatric concern among adolescents, with a prevalence of approximately 5% worldwide (1). In Korea, 13.6% of adolescents report experiencing subjective feelings of sadness or hopelessness for more than 2 weeks in a year (2). As depression is an important predictor of suicide attempts in adolescence (3), predicting and preventing adolescent depression is crucial to public health (4). Moreover, during the coronavirus disease 2019 (COVID-19) pandemic, adolescents have presented with higher levels of anxiety and depression (5), as well as increased risks of suicide (6). Considering the reduced tendency to visit outpatient psychiatric clinics during the COVID-19 pandemic (7), early screening of depression based on predictive factors is even more important.

Depressive symptoms are affected by both genetic and environmental factors (8). Twin and adoption studies have shown that depression tends to be familial, mostly due to genetic influences (9–11). Moreover, a multigenerational family history of depression affects the lifetime prevalence of psychiatric disorders among children (12). Nevertheless, it is necessary not to neglect the importance of environmental effects on the occurrence of depression. One study has suggested that the environment may alter one’s genetic predisposition, whereby it changes the phenotypes of depressive symptoms (13). A recent study reported that high-risk siblings raised in adoptive homes showed a reduced risk of major depression compared with their non-adopted counterparts, suggesting a protective effect of the environment (14).

Diverse environmental factors can affect the development of depression, depending on an individual’s developmental stage (15). In a Korean study, approximately 40% of adolescents who visited an emergency department for self-harm reported their family relationship as the major stressor. Peer relationships, academic stress, and school adaptation may also be considered stressors among adolescents with self-harm behaviors (16). Although various factors can be considered as environmental risk factors for depression, factors that are more associated with the mental health of non-clinically ill adolescents are unclear. Moreover, given that parents play a key role in ensuring the mental health of their children (17), it is important to examine the association of parental factors with the population of non-clinically ill adolescents in Korea.

This study aimed to investigate the socioeconomic factors associated with adolescents’ psychological distress and depression in Korea, using data from the Korea National Health and Nutrition Examination Survey (KNHANES). First, we examined adolescents’ mental health, as well as their drinking and smoking habits. Second, we focused on the relationship between environmental factors and psychological problems among adolescents. Third, we attempted to demonstrate the association of parental factors with adolescent mental health.

## 2. Materials and methods

### 2.1. Study population

**Figure 1:**
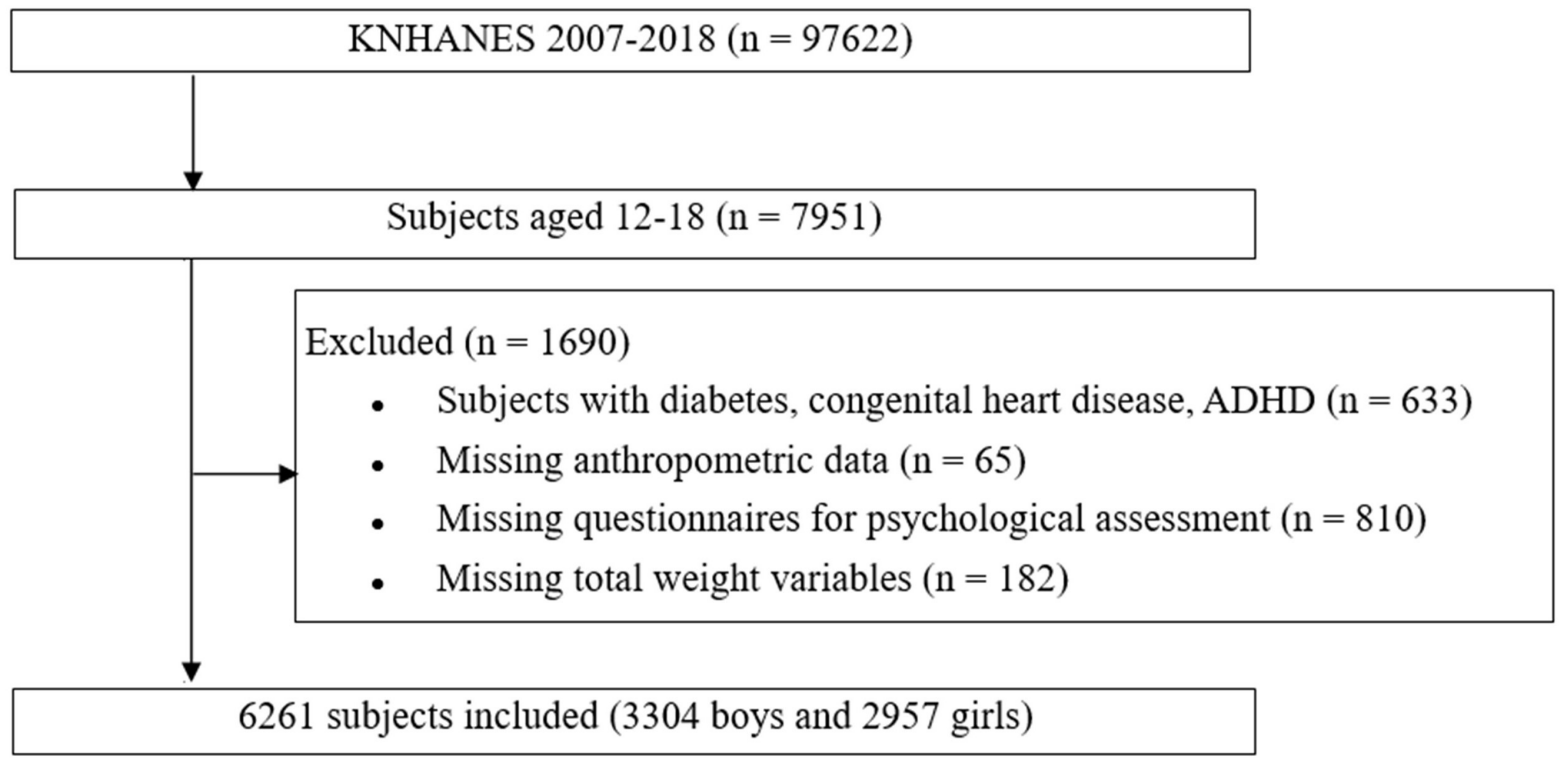
Design and flowchart of study population. Korea National Health and Nutrition Examination Survey (KNHANES); ADHD, attention deficit hyperactivity syndrome.

Of the four phases of KNHANES IV (2007–2009), V (2010–2012), VI (2013–2015), and VII (2016–2018), 6,261 adolescents aged 12–18 years were included in this cross-sectional study. The study design and patient inclusion are illustrated in a flowchart (Figure 1). The KNHANES is a nationally representative cross-sectional survey with a stratified and multistage sampling design that is conducted by the Korea Centers for Disease Control and Prevention based on the National Health Promotion (18). Health interviews, health examinations, and nutrition surveys are included in the survey. Structured interviews were conducted with each participant by trained interviewers and medical technicians. These data provide a variety of information about health status and behavior, socioeconomic demographics, and laboratory test results. Sample weights were used to account for differential probabilities of selection and non-response, and these were included in the estimation processes for all analyses.

### 2.2. Data collection (study variables)

Demographic, anthropometric, and behavioral characteristics were assessed. Height and weight were measured using standard protocols, and body mass index (BMI) was calculated as weight (kg) divided by height squared (m^2^). Height and BMI were presented as standard deviation scores based on the 2017 Korean National Growth Charts (19). Residential districts were classified into urban and rural areas based on administrative districts and populations. Stress perception, depressed mood, and suicidal ideation were assessed to evaluate participants’ psychological distress. Stress perception was assessed using the following question: “How stressed are you on a daily basis?” Responses of “extremely stressed” or “quite stressed” were classified as high stress, and responses of “a little bit stressed” and “hardly stressed” were classified as low stress. Regarding depressed mood, suicidal ideation, suicidal plans, and suicide attempts, participants were asked to answer the following questions (with response options “yes,” “no,” or “I do not know”/no answer): “During the past year, have you felt sad, blue, or depressed nearly every day for two or more weeks?,” “During the past year, have you thought about committing suicide?,” “During the past year, have you seriously planned suicide?,” and “During the past year, have you attempted suicide?,” respectively. These questions are included in the World Health Organization’s Composite International Diagnostic Interview-Short Form, which has been validated as a cost-effective screening method for a general public survey and is a well-documented predictor of suicide attempts that has previously been used in other surveys (20, 21). History of drinking and smoking within the last year as well as sleep duration were investigated using self-reported questionnaires retrospectively.

### 2.3. Statistical analyses

Sampling weights were considered in all analyses to report the representative estimates of the Korean population. Data were analyzed using SAS, version 9.4 (SAS Inc., Cary, NC, United States). All continuous variables were expressed as weighted means with standard errors, whereas categorical variables were expressed as weighted percentages with standard errors. The participants were divided into subgroups according to sex. An independent two-sample t-test and analysis of variance (ANOVA) were used to compare the mean values of the continuous variables and multiple pairwise comparisons for many socioeconomic variables were performed with Bonferroni correction. The Rao-Scott chi-squared test was used to compare the weighted percentage of categorical variables. Univariable logistic regression analyses were performed to investigate the relationships between stress, depressed mood, and suicidal ideation as dependent variables and various markers. In addition, multivariable logistic regression analyses were performed with stepwise selection in which multicolinearity was detected using variance inflation factors. After then, we confirmed that variance inflation factors of all variables were less than 2 in the multivariable model. A *p* value <0.05 was considered statistically significant.

### 2.4. Ethics statement

This study was approved by Yonsei University Gangnam Severance Hospital (IRB, 3–2022-0006).

## 3. Results

### 3.1. Baseline characteristics of the participants

The mean age of the participants was 15.11 years. There were no differences between male and female participants in terms of age, the proportion of rural residence, mean family income, and income below the median. The percentage of drinking among the participants was 26.67%, with its prevalence being significantly higher among males than among females (*p* = 0.001) (Table 1). Smoking was also markedly more prevalent in males (11.74%, *p* < 0.001). Females reported shorter sleep times than males, with an average sleep time of 7.18 h in females (*p* < 0.001). More than a quarter of the adolescents (26.96%) reported high stress, and it was higher among females than among males (*p* < 0.001). Depressed mood and suicidal ideation were reported in 9.55 and 9.2% of adolescents, respectively, and the prevalence of these was higher among females than among males (all *p* < 0.001).

**Table 1:** Baseline characteristics according to sex.

	Total (*n* = 6,261)	Male (*n* = 3,304)	Female (*n* = 2,957)	*p*
Age, y	15.11 (0.03)	15.11 (0.04)	15.11 (0.04)	0.974
Height SDS	0.21 (0.02)	0.23 (0.02)	0.19 (0.02)	0.229
Weight SDS	0.08 (0.02)	0.06 (0.03)	0.10 (0.03)	0.291
BMI SDS	−0.05 (0.02)	−0.09 (0.03)	0.00 (0.03)	0.024
Rural area	15.11 (1.06)	14.84 (1.10)	15.46 (1.27)	0.526
Mean income family, Won	4995.64 (71.93)	4961.94 (81.20)	5034.25 (93.54)	0.464
Income below median	39.16 (0.97)	38.85 (1.16)	39.51 (1.27)	0.643
Mother graduated university	34.55 (0.99)	33.10 (1.18)	36.21 (1.28)	0.031
Father graduated university	46.13 (1.24)	4.16 (1.49)	46.09 (1.55)	0.969
Drinking	26.67 (0.73)	28.83 (0.98)	24.16 (1.02)	0.001*
Drinking mother	75.57 (0.79)	75.37 (1.04)	75.78 (1.02)	0.758
Drinking father	87.97 (0.72)	87.61 (0.91)	88.37 (0.95)	0.521
Smoking	7.94 (0.45)	11.74 (0.74)	3.61 (0.43)	<0.001*
Smoking mother	9.48 (0.55)	9.55 (0.67)	9.40 (0.73)	0.858
Smoking father	83.07 (0.79)	85.36 (0.94)	82.63 (1.07)	0.030
Sleep duration, h/day	7.27 (0.02)	7.34 (0.03)	7.18 (0.03)	<0.001*
High stress	26.96 (0.70)	23.29 (0.88)	31.18 (1.01)	<0.001*
Mother with high stress	28.35 (0.89)	27.77 (1.21)	29.00 (1.14)	0.376
Father with high stress	28.39 (0.98)	28.22 (1.22)	28.59 (1.25)	0.811
Stress				<0.001*
Very low	15.88 (0.55)	17.69 (0.78)	13.81 (0.73)	
Low	57.16 (0.75)	59.03 (1.01)	55.01 (1.06)	
High	23.30 (0.67)	20.43 (0.84)	26.39 (0.96)	
Very high	3.75 (0.29)	2.85 (0.34)	4.79 (0.47)	
Stress of mother				0.387
Very low	9.17 (0.54)	9.85 (0.72)	8.42 (0.69)	
Low	62.48 (0.93)	62.38 (1.19)	62.58 (1.21)	
High	24.46 (0.86)	24.06 (1.09)	24.90 (1.08)	
Very high	3.89 (0.36)	3.71 (0.43)	4.10 (0.51)	
Stress of father				0.117
Very low	11.13 (0.67)	12.28 (0.92)	9.83 (0.80)	
Low	60.48 (1.07)	59.50 (1.35)	61.58 (1.38)	
High	24.31 (0.93)	23.89 (1.14)	24.77 (1.21)	
Very high	4.09 (0.41)	4.33 (0.56)	3.82 (0.52)	
Depression	9.55 (0.46)	7.62 (0.57)	11.77 (0.72)	<0.001*
Mother with depression	13.64 (0.75)	14.08 (0.99)	13.13 (0.92)	0.428
Father with depression	8.30 (0.69)	8.23 (0.84)	8.39 (0.91)	0.881
Suicidal idea	9.20 (0.44)	6.42 (0.52)	12.40 (0.68)	<0.001*
Mother with suicidal idea	11.66 (0.71)	11.49 (0.84)	11.85 (0.97)	0.753
Father with suicidal idea	7.02 (0.62)	7.05 (0.79)	6.98 (0.79)	0.946
Suicidal plan	1.21 (0.24)	0.76 (0.29)	1.69 (0.40)	0.070
Suicidal attempt	1.99 (0.29)	1.50 (0.38)	2.47 (0.43)	0.098
Psychological consultation	4.01 (0.31)	2.99 (0.38)	5.19 (0.51)	<0.001*

SDS, standard deviation score; BMI, body mass index.

Continuous variables are presented as the mean (standard error) and categorical data as the percentage (standard error).

*p* value was corrected using the Bonferroni method for multiple comparisons (*p value < 0.002 (0.05/31)).

### 3.2. Relationship between socioenvironmental factors and adolescents’ mental health

In the depressed mood-specific analysis, adolescents with depressed mood reported a higher prevalence of drinking (36.11%, *p* < 0.001), smoking (14.76%, *p* < 0.001), smoking mothers (15.58%, *p* < 0.001), and less sleep duration (6.92 h/day, *p* < 0.001) than those without depressed mood (Table 2). Adolescents with depressed mood also reported higher levels of stress (66.47%, *p* < 0.001), suicidal ideation (36.70%, *p* < 0.001), suicidal plans (8.16%, *p* < 0.001), and suicidal attempts (9.40%, *p* < 0.001) than those without depressed mood. A higher proportion of mothers of adolescents with depressed mood reported stress (41.05%, *p* < 0.001) and depressed mood (22.93%, *p* < 0.001) than mothers of adolescents without depressed mood.

**Table 2:** Comparison of subjects according to depressed mood.

	Total			Male			Female		
	Yes	No	*p*	Yes	No	*p*	Yes	No	*p*
Age, y	15.50 (0.09)	15.07 (0.03)	<0.001*	15.48 (0.14)	15.08 (0.04)	0.005	15.52 (0.11)	15.05 (0.04)	<0.001*
Height SDS	0.16 (0.06)	0.22 (0.02)	0.268	0.17 (0.08)	0.24 (0.02)	0.446	0.15 (0.07)	0.20 (0.02)	0.463
Weight SDS	0.07 (0.06)	0.08 (0.02)	0.947	−0.00 (0.11)	0.06 (0.03)	0.566	0.13 (0.07)	0.10 (0.03)	0.662
BMI SDS	−0.01 (0.06)	−0.05 (0.02)	0.521	−0.10 (0.10)	−0.08 (0.03)	0.886	0.06 (0.07)	−0.00 (0.03)	0.392
Rural area	14.00 (1.97)	15.20 (1.07)	0.523	14.30 (2.98)	14.85 (1.12)	0.856	13.78 (2.45)	15.61 (1.31)	0.468
Income below median	43.61 (2.50)	38.70 (1.01)	0.053	41.49 (3.96)	38.65 (1.19)	0.481	45.21 (3.18)	38.75 (1.34)	0.050
Mother graduated university	67.10 (2.56)	65.28 (1.03)	0.495	67.97 (3.85)	66.85 (1.24)	0.783	66.40 (3.20)	63.43 (1.35)	0.385
Father graduated university	56.60 (3.08)	53.63 (1.28)	0.343	59.40 (4.66)	53.48 (1.53)	0.218	54.55 (3.98)	53.81 (1.63)	0.858
Drinking	36.11 (2.56)	25.67 (0.75)	<0.001*	36.09 (3.60)	28.24 (1.01)	0.026	36.13 (3.49)	22.58 (1.04)	<0.001*
Drinking mother	76.08 (2.30)	75.57 (0.82)	0.831	75.04 (3.48)	75.46 (1.07)	0.905	76.89 (2.77)	75.70 (1.08)	0.686
Drinking father	86.90 (2.08)	88.06 (0.74)	0.572	84.92 (3.60)	87.79 (0.92)	0.393	88.31 (2.50)	88.38 (1.01)	0.980
Smoking	14.76 (1.79)	7.23 (0.46)	<0.001*	19.30 (3.11)	11.12 (0.75)	0.002	11.32 (2.21)	2.60 (0.38)	<0.001*
Smoking mother	15.58 (2.06)	8.88 (0.55)	<0.001*	15.50 (3.11)	9.08 (0.67)	0.013	15.64 (2.69)	8.65 (0.75)	0.002
Smoking father	81.89 (2.50)	84.27 (0.81)	0.329	82.74 (3.41)	85.57 (0.96)	0.387	81.27 (3.16)	82.74 (1.13)	0.649
Sleep duration, h/day	6.92 (0.07)	7.31 (0.02)	<0.001*	7.04 (0.10)	7.37 (0.03)	0.002	6.83 (0.09)	7.23 (0.04)	<0.001*
High stress	66.47 (2.30)	22.79 (0.68)	<0.001*	61.56 (3.64)	20.13 (0.87)	<0.001*	70.12 (2.90)	25.99 (1.01)	<0.001*
Mother with high stress	41.05 (2.51)	27.03 (0.92)	<0.001*	41.51 (3.99)	26.62 (1.15)	<0.001*	40.69 (3.26)	27.52 (1.19)	<0.001*
Father with high stress	24.75 (2.55)	28.76 (1.02)	0.145	20.24 (3.58)	28.89 (1.26)	0.036	28.01 (3.61)	28.62 (1.33)	0.875
Stress			<0.001*			<0.001*			<0.001*
Very low	2.58 (0.80)	17.29 (0.58)		5.30 (1.80)	18.71 (0.83)		0.56 (0.34)	15.58 (0.82)	
Low	30.95 (2.24)	59.92 (0.76)		33.14 (3.52)	61.16 (1.04)		29.32 (2.90)	58.43 (1.11)	
High	49.69 (2.53)	20.41 (0.65)		51.20 (3.86)	17.89 (0.83)		48.56 (3.27)	23.44 (0.96)	
Very high	16.78 (1.78)	2.38 (0.24)		10.36 (2.03)	2.23 (0.32)		21.57 (2.63)	2.55 (0.37)	
Stress of mother			<0.001*			0.001*			<0.001*
Very low	6.11 (1.28)	9.49 (0.57)		7.69 (2.31)	10.04 (0.76)		4.88 (1.36)	8.85 (0.75)	
Low	52.83 (2.51)	63.47 (0.97)		50.79 (3.85)	63.34 (1.23)		54.43 (3.26)	63.63 (1.27)	
High	33.33 (2.43)	23.53 (0.88)		36.23 (3.86)	23.03 (1.11)		31.06 (3.08)	24.10 (1.14)	
Very high	7.73 (1.57)	3.51 (0.35)		5.28 (2.10)	3.58 (0.43)		9.63 (2.24)	3.42 (0.47)	
Stress of father			0.538			0.150			0.866
Very low	11.10 (1.97)	11.08 (0.70)		11.48 (3.03)	12.30 (0.96)		10.83 (2.50)	9.65 (0.83)	
Low	64.14 (2.89)	60.16 (1.11)		68.28 (4.29)	58.81 (1.40)		61.15 (3.95)	61.74 (1.48)	
High	21.18 (2.30)	24.65 (0.97)		15.63 (3.15)	24.60 (1.19)		25.19 (3.31)	24.70 (1.30)	
Very high	3.57 (1.09)	4.12 (0.43)		4.61 (2.04)	4.29 (0.57)		2.82 (1.16)	3.92 (0.56)	
Mother with depression	22.93 (2.64)	12.67 (0.75)	<0.001*	20.52 (3.76)	13.55 (1.01)	0.036	24.85 (3.58)	11.62 (0.88)	<0.001*
Father with depression	9.81 (1.93)	8.07 (0.72)	0.356	5.53 (2.06)	8.43 (0.89)	0.262	13.13 (2.91)	7.64 (0.94)	0.033
Suicidal idea	36.70 (2.29)	6.30 (0.39)	<0.001*	30.96 (3.48)	4.40 (0.44)	<0.001*	40.97 (3.00)	8.59 (0.62)	<0.001*
Mother with suicidal idea	17.36 (2.38)	11.06 (0.72)	0.003	19.06 (3.64)	10.85 (0.85)	0.008	16.00 (3.12)	11.32 (1.00)	0.103
Father with suicidal idea	9.34 (2.17)	6.79 (0.63)	0.197	5.67 (2.35)	7.18 (0.83)	0.578	12.19 (3.31)	6.31 (0.76)	0.024
Suicidal plan	8.16 (2.10)	0.56 (0.18)	<0.001*	7.24 (3.53)	0.33 (0.19)	<0.001*	8.73 (2.63)	0.82 (0.31)	<0.001*
Suicidal attempt	9.40 (1.68)	0.98 (0.22)	<0.001*	11.35 (3.28)	0.54 (0.24)	<0.001*	8.24 (1.89)	1.45 (0.37)	<0.001*
Psychological consultation	16.09 (1.77)	2.49 (0.26)	<0.001*	12.99 (2.58)	2.00 (0.32)	<0.001*	18.40 (2.44)	3.07 (0.44)	<0.001*

SDS, standard deviation score; BMI, body mass index.

Continuous variables are presented as the mean (standard error) and categorical data as the percentage (standard error).

*p* value was corrected using the Bonferroni method for multiple comparisons (*p value < 0.002 (0.05/29)).

In the subgroup analysis according to stress perception, adolescents with higher stress also showed higher BMI SDS (0.07, *p* < 0.001) and reported a higher proportion of low-income parents (42.09%, *p* = 0.022), a higher proportion of drinking history (30.19%, *p* < 0.001), and a higher proportion of mothers with depressed mood (16.32%, *p* = 0.012) compared to those with lower stress (Supplementary Table S1). This tendency differed by sex, with only females showing a similar tendency. Moreover, regardless of sex, adolescents with higher stress reported higher proportions of smoking (10.48%, *p* < 0.001), smoking mothers (12.81%, *p* < 0.001), mothers with high stress (33.35%, *p* < 0.001), and less sleep duration (7.37 h/day, *p* < 0.001) than those with lower stress.

Concerning suicidal ideation, adolescents with experience of suicidal ideation reported a higher proportion of drinking (35.92%, *p* < 0.001), smoking (11.53%, *p* = 0.009), and smoking mothers (14.60%, *p* < 0.001), a lower proportion of smoking fathers (79.30%, *p* = 0.041), and shorter sleep duration (6.84 h/day, *p* < 0.001) compared to those without suicidal ideation (Supplementary Table S2). Adolescents with suicidal ideation reported higher stress (65.40%, *p* < 0.001) and higher maternal stress (36.71%, *p* < 0.001), but not with higher paternal stress (*p* = 0.344), compared to those without. Adolescents with suicidal ideation also reported a higher prevalence of depressed mood (38.10%), mothers with depressed mood (18.67%), and fathers with depressed mood (19.85%) than those without (all *p* < 0.001).

### 3.3. Relationship between the mental health of mothers and the mental health of adolescents

In the subgroup analysis according to maternal stress, high stress (31.32%, *p* < 0.001), depressed mood (13.45%, *p* < 0.001), suicidal ideation (11.62%, *p* < 0.001), suicidal plans (1.87%, *p* = 0.037), and suicide attempts (2.68%, *p* = 0.033) were higher in participants whose mothers had higher stress than that in those whose mothers did not have as high stress (Table 3). These tendencies related to maternal stress were more obvious in females than in males. In females, the prevalence of drinking (29.45%, *p* < 0.001) and smoking (5.26%, *p* = 0.004) was higher in participants with higher maternal stress than that in those without.

**Table 3:** Comparison of the subjects according to mother’s stress.

	Total			Male			Female		
	Low stress	High stress	*p*	Low stress	High stress	*p*	Low stress	High stress	*p*
Drinking	24.62 (0.91)	28.38 (1.40)	0.021	27.76 (1.26)	27.38 (1.89)	0.865	21.02 (1.21)	29.45 (2.03)	<0.001*
Smoking	6.54 (0.52)	8.59 (0.91)	0.041	10.09 (0.89)	11.68 (1.40)	0.322	2.50 (0.43)	5.26 (1.05)	0.004*
High stress	24.76 (0.84)	31.32 (1.46)	<0.001*	21.25 (1.07)	27.83 (1.90)	0.002*	28.78 (1.21)	35.09 (2.07)	0.007
Stress			<0.001*			0.011			0.009
Very low	17.01 (0.70)	13.89 (1.06)		18.91 (1.03)	15.60 (1.54)		14.83 (0.93)	12.04 (1.47)	
Low	58.23 (0.94)	54.79 (1.56)		59.84 (1.27)	56.57 (2.09)		56.39 (1.32)	52.87 (2.20)	
High	21.68 (0.83)	26.46 (1.38)		18.82 (1.05)	24.70 (1.85)		24.96 (1.18)	28.37 (1.95)	
Very high	3.08 (0.32)	4.86 (0.64)		2.43 (0.38)	3.13 (0.70)		3.82 (0.51)	6.72 (1.11)	
Depression	7.64 (0.49)	13.45 (1.04)	<0.001*	6.22 (0.64)	11.48 (1.39)	<0.001*	9.26 (0.78)	15.57 (1.54)	<0.001*
Suicidal idea	7.92 (0.50)	11.62 (0.96)	<0.001*	5.79 (0.63)	7.60 (1.12)	0.143	10.37 (0.79)	15.94 (1.57)	0.001*
Suicidal plan	0.73 (0.26)	1.87 (0.56)	0.037	0.84 (0.41)	0.54 (0.34)	0.578	0.60 (0.31)	3.14 (1.04)	0.003*
Suicidal attempt	1.35 (0.30)	2.68 (0.63)	0.033	1.13 (0.42)	1.48 (0.75)	0.661	1.58 (0.45)	3.70 (1.01)	0.028
Psychological consultation	3.05 (0.34)	5.90 (0.79)	<0.001*	2.36 (0.42)	3.81 (0.88)	0.100	3.83 (0.56)	8.17 (1.27)	<0.001*

Values are presented as the percentage (standard error).

*p* value was corrected using the Bonferroni method for multiple comparisons (*p value < 0.006 (0.05/9)).

**Figure 2:**
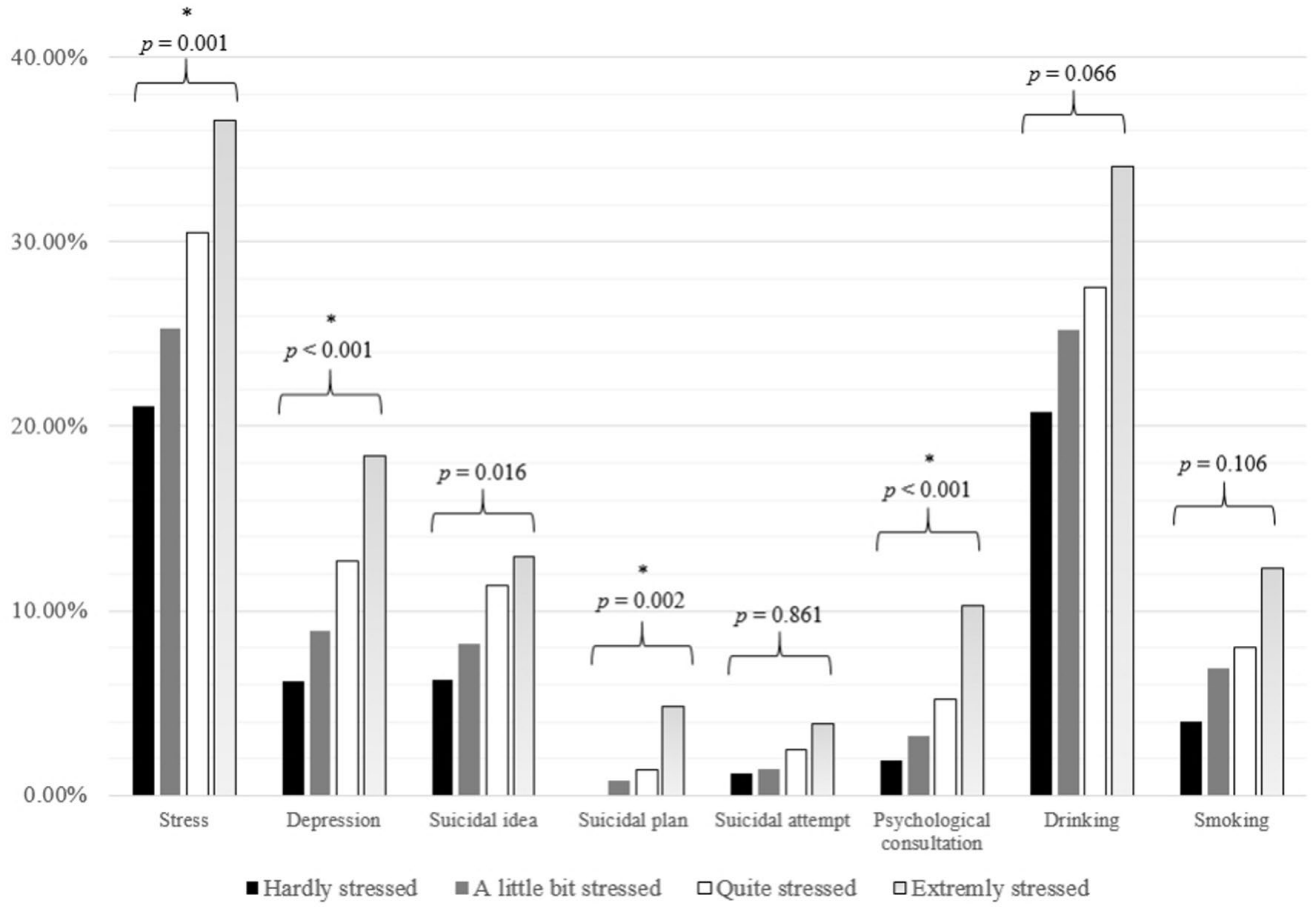
Psychological distress according to maternal stress among participants. The Bonferroni corrected *p* value of each analysis of variance is presented above the bars. *p* value was corrected using the Bonferroni method for multiple comparisons (*p value <0.006 (0.05/8)).

In the ANOVA by maternal stress, the prevalence of stress, depressed mood, suicidal ideation, and psychological consultation in adolescents increased with an increase in maternal stress level (Figure 2).

Maternal depressed mood was associated with a higher prevalence of drinking (33.75%, *p* < 0.001), smoking (12.67%, *p* < 0.01), high stress (32.21%, *p* = 0.012), depressed mood (15.95%, *p* < 0.001), suicidal ideation (14.34%, *p* = 0.006), and suicidal plans (2.61%, *p* = 0.011) (Supplementary Table S3). Likewise, the prevalence of drinking (31.69%, *p* = 0.009), smoking (11.62%, *p* = 0.002), high stress (31.63%, *p* = 0.032), depressed mood (14.13%, *p* = 0.003), suicidal ideation (17.84%, *p* < 0.001), and suicidal plans (5.05%, *p* = 0.001) were higher among adolescents whose mothers reported suicidal ideation (Supplementary Table S4).

### 3.4. Risk factors for stress, depressed mood, and suicidal idea by logistic regression analysis

In the univariable logistic regression analysis for depressed mood, the odds ratios (ORs) and 95% confidence intervals (CIs) were significantly higher for drinking (OR = 1.637, 95% CI: 1.300–2.061), smoking (OR = 2.221, 95% CI: 1.634–3.019), high stress (OR = 6.717, 95% CI: 5.432–8.306), and suicidal ideation (OR = 8.626, 95% CI: 6.804–10.938) in the total population, as well as among male and female adolescents (Table 4). Moreover, smoking mothers (OR = 1.894, 95% CI: 1.370–2.619), mothers with high stress (OR = 1.880, 95% CI: 1.518–2.328), and mothers with depressed mood (OR = 2.051, 95% CI: 1.503–2.797) showed high ORs for depressed mood in the total population, as well as among both male and female adolescents. The high OR for mothers with suicidal ideation (OR = 1.689, 95% CI: 1.199–2.379) was significant only in the total population and among male adolescents. Sleep duration (OR = 0.830, 95% CI: 0.775–0.887) showed a lower OR in the total population, as well as among both male and female adolescents. In multivariable logistic regression with stepwise selection for depressed mood, the ORs were significantly higher for age, stress, mothers with depressed mood, and fathers with depressed mood among the total population and stress, mothers with depressed mood, and fathers with depressed mood among male and female adolescents (Table 5).

**Table 4:** Logistic regression for depression.

	Total		Male		Female	
	OR (95% CI)	*p*	OR (95% CI)	*p*	OR (95% CI)	*p*
Age, y	1.125 (1.070–1.184)	<0.001	1.114 (1.031–1.205)	0.007	1.134 (1.0610–1.213)	<0.001
Area						
Urban	ref		ref		ref	
Rural	0.909 (0.677–1.220)	0.524	0.957 (0.597–1.535)	0.856	0.864 (0.582–1.283)	0.468
Income						
Below median	ref		ref		ref	
Above median	0.816 (0.665–1.003)	0.053	0.888 (0.639–1.235)	0.482	0.767 (0.587–1.001)	0.051
Mother education						
-High school	ref		ref		ref	
University-	0.922 (0.729–1.165)	0.496	0.950 (0.661–1.367)	0.783	0.878 (0.654–1.1780)	0.385
Father education						
-High school	ref		ref		ref	
University-	0.887 (0.692–1.137)	0.344	0.786 (0.535–1.155)	0.219	0.970 (0.698–1.350)	0.858
Drinking	1.637 (1.300–2.061)	<0.001	1.435 (1.041–1.979)	0.027	1.940 (1.413–2.665)	<0.001
Drinking mother	1.028 (0.797–1.326)	0.832	0.977 (0.670–1.425)	0.905	1.068 (0.775–1.472)	0.687
Drinking father	0.900 (0.623–1.299)	0.572	0.784 (0.448–1.372)	0.394	0.994 (0.598–1.650)	0.980
Smoking	2.221 (1.634–3.019)	<0.001	1.913 (1.262–2.899)	0.002	4.775 (2.823–8.078)	<0.001
Smoking mother	1.894 (1.370–2.619)	<0.001	1.837 (1.130–2.987)	0.014	1.959 (1.263–3.037)	0.003
Smoking father	0.844 (0.600–1.186)	0.329	0.809 (0.500–1.309)	0.387	0.905 (0.589–1.391)	0.650
Sleep duration, h/day	0.830 (0.775–0.887)	<0.001	0.844 (0.757–0.942)	0.002	0.833 (0.763–0.910)	<0.001
High stress	6.717 (5.432–8.306)	<0.001	6.354 (4.612–8.756)	<0.001	6.685 (5.001–8.937)	<0.001
Stress						
Very low	ref		ref		ref	
Low	3.459 (1.823–6.560)	<0.001	1.912 (0.913–4.005)	<0.001	14.057 (4.083–48.400)	0.0333
High	16.303 (8.650–30.725)	<0.001	10.096 (4.895–20.826)	<0.001	58.042 (16.991–198.272)	<0.001
Very high	47.275 (23.816–93.841)	<0.001	16.355 (7.011–38.156)	<0.001	237.011 (66.1845848.753)	<0.001
Mother with high stress	1.880 (1.518–2.328)	<0.001	1.957 (1.395–2.746)	<0.001	1.807 (1.360–2.402)	<0.001
Father with high stress	0.815 (0.618–1.074)	0.146	0.624 (0.400–0.974)	0.038	0.971 (0.669–1.409)	0.876
Stress of mother						
Very low	ref		ref		ref	
Low	1.293 (0.822–2.033)	0.266	1.047 (0.540–2.028)	0.892	1.551 (0.853–2.820)	0.150
High	2.200 (1.379–3.511)	0.040	2.054 (1.014–4.161)	0.037	2.337 (1.251–4.366)	0.397
Very high	3.422 (1.828–6.406)	<0.001	1.924 (0.665–5.570)	0.372	5.113 (2.323–11.253)	<0.001
Stress of father						
Very low	ref		ref		ref	
Low	1.064 (0.700–1.616)	0.305	1.244 (0.668–2.317)	0.195	0.882 (0.509–1.528)	0.803
High	0.858 (0.543–1.353)	0.460	0.680 (0.332–1.3930)	0.083	0.908 (0.504–1.635)	0.666
Very high	0.865 (0.411–1.821)	0.729	1.151 (0.390–3.398)	0.687	0.642 (0.234–1.756)	0.407
Mother with depression	2.051 (1.503–2.797)	<0.001	1.647 (1.029–2.636)	0.038	2.515 (1.689–3.745)	<0.001
Father with depression	1.239 (0.785–1.956)	0.357	0.636 (0.286–1.412)	0.266	1.828 (1.042–3.204)	0.035
Suicidal idea	8.626 (6.804–10.938)	<0.001	9.755 (6.690–14.225)	<0.001	7.387 (5.552–9.830)	<0.001
Mother with suicidal idea	1.689 (1.199–2.379)	0.003	1.936 (1.186–3.160)	0.003	1.492 (0.921–2.417)	0.104
Father with suicidal idea	1.414 (0.832–2.403)	0.200	0.777 (0.318–1.897)	0.579	2.059 (1.082–3.920)	0.028

OR, odds ratio; CI, confidence interval.

**Table 5:** Multivariable logistic regression with stepwise selection for depressed mood.

	Total		Male		Female	
	OR (95% CI)	*p*	OR (95% CI)	*p*	OR (95% CI)	*p*
Age, y	1.085 (1.010-1.166)	0.025				
Stress						
Very low	ref		ref		ref	
Low	3.569 (1.657-7.686)	0.001	1.683 (0.806-3.516)	0.166	8.138 (2.367-27.986)	0.001
High	9.481 (4.335-20.735)	<0.001	6.831 (3.263-14.300)	<0.001	19.376 (5.590-67.168)	<0.001
Very high	28.034 (11.988-65.560)	<0.001	9.404 (3.848-22.986)	<0.001	77.812 (21.139-286.421)	<0.001
Mother with depressed mood	1.785 (1.275-2.499)	0.001			1.936 (1.232-3.043)	0.004
Father with depressed mood	5.445 (4.015-7.384)	<0.001	5.673 (3.686-8.733)	<0.001	4.784 (3.242-7.058)	<0.001
Suicidal ideation						

OR, odds ratio; CI, confidence interval.

In the univariable logistic regression analysis for stress, ORs were significantly higher for smoking (OR = 1.553, 95% CI: 1.214–1.998), smoking mothers (OR = 1.624, 95% CI: 1.284–2.054) and mothers with high stress (OR = 1.3860, 95% CI: 1.1834–1.6232) in the total adolescent population as well as in both male and female groups (Supplementary Table S5). The ORs for stress were significant in the total population and among female adolescents who drank (OR = 1.2726, 95% CI: 1.1040–1.4668) and who had mothers with depressed mood (OR = 1.3462, 95% CI: 1.0663–1.6994). Meanwhile, income above the median (OR = 0.8467, 95% CI: 0.7345–0.9760) and sleep duration (OR = 0.8255, 95% CI: 0.7854–0.8677) showed significantly lower ORs in the total population. In multivariable logistic regression with stepwise selection for stress, ORs were significantly higher for smoking mothers, mother with stress, depressed mood, and suicidal ideation, and they were lower for income above median and sleep duration among the total population (Supplementary Table S6). The corresponding values were significantly higher for smoking, smoking mothers, mothers with stress, depressed mood, and suicidal ideation and lower for sleep duration among male adolescents. Among the female adolescents, the corresponding values were significantly higher for smoking, fathers with stress, depressed mood, and suicidal ideation and lower for sleep duration.

In univariable logistic regression analysis for suicidal ideation, the ORs of drinking (OR = 1.619, 95% CI: 1.313–1.995), smoking (OR = 1.589, 95% CI: 1.121–2.253), smoking mothers (OR = 1.729, 95% CI: 1.254–2.384), mothers with high stress (OR = 1.528, 95% CI: 1.219–1.915), mothers with depressed mood (OR = 1.530, (%%CI: 1.126–2.079), fathers with depressed mood (OR = 1.530, 95% CI: 1.126–2.079), mothers with suicidal ideation (OR = 2.067, 95% CI: 1.487–2.872), and fathers with suicidal ideation (OR = 2.067, 95% CI: 1.487–2.872) were significantly higher in the total population (Supplementary Table S7). Sleep duration and smoking fathers (OR = 0.703, 95% CI: 0.501–0.987) were negatively associated with suicidal ideation (OR = 0.793, 95% CI: 0.736–0.855). In the multivariable logistic regression with stepwise selection for suicidal ideation, the ORs were significantly higher for stress, depressed mood, mothers with suicidal ideation, and fathers with suicidal ideation and lower for smoking fathers and sleep duration for the total population (Supplementary Table S8). The corresponding values were significantly higher for high stress and depressed mood and lower for smoking fathers and sleep duration among male adolescents. Among female adolescents, the corresponding values were significantly higher for stress, depressed mood, mothers with suicidal ideation, and fathers with suicidal ideation.

## 4. Discussion

In addition to depressed mood and suicidal ideation among mothers, a higher perception of maternal stress was related to higher levels of stress perception, depressed mood, and suicidal ideation among adolescents. However, the association of adolescents’ mental health with fathers’ mental health was weaker than that with mothers’ mental health. Additionally, a higher prevalence of smoking and drinking was commonly reported in adolescents with higher stress perception, depressed mood, and suicidal ideation. A high proportion of low income was commonly reported in adolescents with stress, especially in females.

More than 10% of adolescents reported ≥2 weeks of depressed mood in a year, with female adolescents showing a higher prevalence. This result is in line with a previous report suggesting that 13.6% of adolescents experience depressed moods for ≥2 weeks in Korea (2). Although some adolescents may not be diagnosed with major depressive disorders according to the Diagnostic and Statistical Manual of Mental Disorders (DSM-5) (22) the prevalence of experience of depressed mood is twice as prevalent as adolescent depression (1). Considering that the median age of a major depressive disorder onset is about 25 years (23), the onset of depressive symptoms at age 15 years may implicate adolescents’ distinguishing risk factors for depression as either genetic or environmental (13, 24). Moreover, depression in early adolescence tends to recur in late adolescence or early adulthood (25), especially in those with higher severity of depression (26). Adolescents with depressive symptoms need to be carefully monitored for biological, psychological, and environmental risk factors.

In our study, poor maternal mental health (high stress perception, depressed mood, and suicidal ideation) was associated with mental health problems (high stress perception, depressed mood, and suicidal ideation) in adolescents. Among various risk factors affecting adolescent depression (8), parental factors are closely related to adolescent depression (27). About 20% of children are influenced by parental mental health, and their probability of developing mental illness is up to 50% compared to other children (28). In particular, it has been established that maternal depression is strongly associated with children’s psychopathology, including depression (29). Despite reports showing a significant relationship between paternal and adolescent depression (30), our results showed an inconsistent relationship between fathers’ and adolescents’ mental health.

There are several explanations for the relationship between maternal depression and adolescent depression (29). Concurrent maternal depression may affect adolescents more environmentally than inherently (31), suggesting the importance of proper management of maternal depression in preventing adolescent depression. Moreover, while maternal depression has been suggested as an important predictor of adolescent depression (32), the mechanism may be both direct and indirect (32). Maternal depression influences the social and emotional functioning of adolescents, as adolescents with depressive mothers are easily exposed to emotion regulation difficulties (33), resulting in poor relationships with their mothers (34) and poor interpersonal function (35). Poor interpersonal function may develop emotional vulnerability in adolescents (36). We believe that this indirect pathway of maternal depression to adolescent depression would also be seen in mothers with higher stress perception, resulting in adolescents’ poor mental health. Conflicts with parents are associated with not only clinically diagnosed depression but also subclinical depression in adolescents (37). According to the stress-buffering hypothesis, closeness with the mother is an important buffering factor in experiencing stressful life events (38). Considering that adolescent depression could also influence parental mental health (39), the mental health relationship between mothers and adolescents in our study seems to be mutual. The quality of the relationship appears to play an important role in their interaction (34). Further studies are required to clarify casualty between parental mental health and that of adolescents.

In our study, the prevalence of drinking and smoking behaviors was higher among adolescents with depressed moods. It is well known that adolescent depression and psychological distress are related to smoking and drinking behaviors (40, 41). Furthermore, alcohol consumption and smoking behaviors are bi-directionally associated with adolescent depression (42, 43). Smoking and drinking behaviors have important clinical implications, as they suggest a higher possibility of suicidal behavior (44, 45). Similarly, our results showed an association between smoking and drinking behaviors and suicidal ideation in adolescents. Thus, close monitoring of smoking and drinking behaviors for adolescents with mental health problems, early identification, and strengthening of interventions for mental health promotion for those with smoking and/or drinking habits is required, taking into consideration their parents’ mental health.

Low-income status was related to stress in our study. A systematic review reported that low socioeconomic status is a risk factor for mental health problems among children and adolescents (46). As a family’s income usually comes from parents and the income status is related to parental socioeconomic status rather than that of adolescents, it impacts both adolescent and parental stress. This can negatively affect the adolescent-parent connection, which is a protective factor for adolescent mental health. Thus, the results indicate the necessity for proper interventions for support of families support with low incomes in public policy programs.

Our study had several limitations. First, this study was based on a large-scale adolescent survey; thus, it was impossible to conduct structured interviews or measurement instruments for depression such as the Beck Depression Inventory to diagnose major depressive disorders. Given that the experience of depressed mood was estimated using a questionnaire, the responses of adolescents could be subjective, and answers may not exactly match clinical major depressive disorders. Self-reported items cannot represent all of adolescents’ mental health. Second, the cross-sectional nature of this study precludes causal inferences about the relationship between socio-environmental factors and mental health problems.

In conclusion, our study revealed that high perception of maternal stress as well as depressed mood and suicidal ideation are significantly associated with adolescents’ mental health experiences (experience of depressed mood and suicidal ideation, high perception of stress) using large-scale data from a non-clinically ill adolescent population in Korea. Our results suggest the role of maternal mental health as an environmental factor related to adolescents’ mental health. Moreover, smoking and alcohol consumption are associated with mental health problems, including high stress perception, depressed mood, and suicidal ideation. Further, low-income status was related to stress. It is necessary to closely monitor adolescents’ mental health, as well as smoking and drinking behaviors, especially those whose mothers also present with mental health problems, and/or those in low-income family environments, and/or those whose parents exhibit smoking and/or drinking behaviors.

## Data availability statement

The original contributions presented in the study are included in the article/Supplementary materials, further inquiries can be directed to the corresponding author.

## Ethics statement

The studies involving human participants were reviewed and approved by Institutional Review Board at Yonsei University Gangnam Severance Hospital. Written informed consent from the participants’ legal guardian/next of kin was not required to participate in this study in accordance with the national legislation and the institutional requirements.

## Author contributions

KS, JL, SL, and HC: conceptualization, formal analysis, and investigation. SJ: data curation. KS, JL, HL, SL, and HC: methodology. JL: writing-original draft. KS, SJ, HL, SL, H-SK, and HC: writing-review and editing. H-SK: supervision. All authors contributed to the article and approved the submitted version.

## Conflict of interest

The authors declare that the research was conducted in the absence of any commercial or financial relationships that could be construed as a potential conflict of interest.

## Publisher’s note

All claims expressed in this article are solely those of the authors and do not necessarily represent those of their affiliated organizations, or those of the publisher, the editors and the reviewers. Any product that may be evaluated in this article, or claim that may be made by its manufacturer, is not guaranteed or endorsed by the publisher.
