# Supplementary material for: Association of maternal mental health and drinking/smoking with adolescents’ mental health based on the Korea National Health and Nutrition Examination Survey
Source: Front Psychiatry. 2023 Jun 20;14:1087300. doi: 10.3389/fpsyt.2023.1087300 (PMC10321712; doi:10.3389/fpsyt.2023.1087300)
Supplement: Supplementary file 1 [file Table_1.DOCX]

Supplementary Table 1. Comparison of the subjects according to stress

|  | Total | |  | Male | |  | Female | |  |
| --- | --- | --- | --- | --- | --- | --- | --- | --- | --- |
|  | Low stress | High stress | *p* | Low stress | High stress | *p* | Low stress | High stress | *p* |
| Age, y | 15.03 (0.03) | 15.31 (0.05) | <0.001 | 15.08 (0.04) | 15.02 (0.08) | 0.167 | 14.97 (0.05) | 15.40 (0.07) | <0.001 |
| Height SDS | 0.22 (0.02) | 0.19 (0.03) | 0.372 | 0.24 (0.03) | 0.20 (0.04) | 0.419 | 0.20 (0.03) | 0.19 (0.04) | 0.734 |
| Weight SDS | 0.05 (0.02) | 0.16 (0.04) | 0.0109 | 0.04 (0.03) | 0.11 (0.06) | 0.2781 | 0.055 (0.03) | 0.198 (0.05) | 0.013 |
| BMI SDS | -0.09 (0.02) | 0.07 (0.04) | <0.001 | -0.11 (0.03) | 0.00 (0.06) | 0.0926 | -0.053 (0.03) | 0.129 (0.05) | 0.002 |
| Rural area | 15.54 (1.13) | 13.82 (1.38) | 0.182 | 15.11 (1.14) | 13.82 (1.65) | 0.419 | 16.11 (1.40) | 13.82 (1.64) | 0.179 |
| Income below median | 38.10 (1.08) | 42.09 (1.61) | 0.022 | 38.39 (1.29) | 40.44 (2.17) | 0.396 | 37.72 (1.48) | 43.52 (2.04) | 0.014 |
| Mother graduated university | 34.14 (1.07) | 35.74 (1.71) | 0.366 | 32.31 (1.29) | 35.60 (2.30) | 0.1821 | 36.45 (1.48) | 35.87 (2.15) | 0.813 |
| Father graduated university | 46.37 (1.35) | 45.35 (2.11) | 0.651 | 45.97 (1.61) | 46.46 (2.93) | 0.8757 | 46.86 (1.81) | 44.41 (2.52) | 0.403 |
| Drinking | 25.37 (0.79) | 30.19 (1.38) | <0.001 | 28.33 (1.07) | 30.50 (2.01) | 0.316 | 21.57 (1.14) | 29.92 (1.87) | <0.001 |
| Drinking mother | 75.25 (0.89) | 76.62 (1.37) | 0.375 | 75.29 (1.15) | 75.89 (1.99) | 0.785 | 75.21 (1.21) | 77.23 (1.75) | 0.330 |
| Drinking father | 87.54 (0.82) | 89.11 (1.15) | 0.240 | 87.29 (1.02) | 88.57 (1.70) | 0.511 | 87.86 (1.15) | 89.57 (1.44) | 0.339 |
| Smoking | 7.01 (0.50) | 10.48 (0.96) | <0.001 | 10.41 (0.79) | 16.11 (1.74) | 0.001 | 2.70 (0.46) | 5.65 (0.95) | 0.002 |
| Smoking mother | 8.30 (0.59) | 12.81 (1.12) | <0.001 | 8.46 (0.70) | 13.27 (1.59) | 0.002 | 8.10 (0.84) | 12.42 (1.45) | 0.005 |
| Smoking father | 83.76 (0.88) | 84.89 (1.42) | 0.479 | 85.44 (1.05) | 85.12 (1.98) | 0.885 | 81.66 (1.27) | 84.68 (1.83) | 0.174 |
| Sleep duration, h/day | 7.37 (0.03) | 6.99 (0.05) | <0.001 | 7.416 (0.03) | 7.105 (0.07) | <0.001 | 7.320 (0.04) | 6.879 (0.06) | <0.001 |
| Mother with high stress | 26.53 (1.02) | 33.35 (1.52) | <0.001 | 26.05 (1.24) | 33.47 (2.22) | 0.002 | 27.13 (1.34) | 33.25 (1.97) | 0.007 |
| Father with high stress | 27.57 (1.08) | 30.77 (1.77) | 0.094 | 28.27 (1.35) | 28.26 (2.47) | 0.996 | 26.70 (1.46) | 32.94 (2.27) | 0.016 |
| Stress of mother |  |  | <0.001 |  |  | 0.016 |  |  | 0.007 |
| Very low | 9.87 (0.62) | 7.28 (0.95) |  | 10.24 (0.80) | 8.60 (1.51) |  | 9.41 (0.87) | 6.15 (0.97) |  |
| Low | 63.60 (1.06) | 59.37 (1.60) |  | 63.71 (1.32) | 57.93 (2.34) |  | 63.46 (1.41) | 60.59 (2.05) |  |
| High | 23.16 (0.94) | 27.99 (1.44) |  | 22.81 (1.20) | 28.17 (2.07) |  | 23.60 (1.25) | 27.84 (1.90) |  |
| Very high | 3.37 (0.41) | 5.37 (0.73) |  | 3.24 (0.45) | 5.30 (1.09) |  | 3.53 (0.60) | 5.42 (0.97) |  |
| Stress of father |  |  | 0.302 |  |  | 0.607 |  |  | 0.103 |
| Very low | 11.19 (0.76) | 10.78 (1.16) |  | 11.98 (1.01) | 13.11 (1.88) |  | 10.21 (0.97) | 8.78 (1.32) |  |
| Low | 61.24 (1.19) | 58.44 (1.84) |  | 59.75 (1.48) | 58.63 (2.73) |  | 63.08 (1.61) | 58.29 (2.41) |  |
| High | 23.82 (1.03) | 25.84 (1.68) |  | 24.29 (1.29) | 22.83 (2.26) |  | 23.22 (1.41) | 28.43 (2.18) |  |
| Very high | 3.76 (0.44) | 4.94 (0.84) |  | 3.98 (0.58) | 5.43 (1.33) |  | 3.48 (0.57) | 4.51 (1.00) |  |
| Depressed mood | 4.39 (0.36) | 23.55 (1.26) | <0.001 | 3.82 (0.46) | 20.15 (1.78) | <0.0001 | 5.11 (0.60) | 26.48 (1.68) | <0.001 |
| Mother with depressed mood | 12.66 (0.84) | 16.32 (1.38) | 0.012 | 13.43 (1.08) | 16.34 (2.05) | 0.1747 | 11.66 (1.06) | 16.31 (1.71) | 0.014 |
| Father with depressed mood | 7.84 (0.78) | 9.38 (1.20) | 0.237 | 7.72 (0.90) | 9.83 (1.81) | 0.2509 | 8.00 (1.10) | 8.99 (1.49) | 0.580 |
| Suicidal ideation | 4.36 (0.35) | 22.32 (1.20) | <0.001 | 3.09 (0.39) | 17.38 (1.69) | <0.0001 | 5.98 (0.59) | 26.56 (1.69) | <0.001 |
| Mother with suicidal ideation | 10.90 (0.79) | 13.72 (1.24) | 0.032 | 10.77 (0.92) | 13.93 (1.82) | 0.0932 | 11.08 (1.13) | 13.54 (1.55) | 0.156 |
| Father with suicidal ideation | 6.46 (0.70) | 8.57 (1.07) | 0.068 | 6.66 (0.90) | 8.41 (1.62) | 0.3245 | 6.21 (0.92) | 8.72 (1.37) | 0.102 |
| Suicidal plan | 0.13 (0.13) | 4.25 (0.85) | <0.001 | 0.23 (0.22) | 2.62 (1.03) | 0.0039 | 0.00 (0.00) | 5.54 (1.30) | <0.001 |
| Suicidal attempt | 0.50 (0.19) | 5.32 (0.80) | <0.001 | 0.60 (0.28) | 4.11 (1.21) | <0.0001 | 0.38 (0.24) | 6.18 (1.11) | <0.001 |
| Psychological consultation | 2.04 (0.27) | 8.98 (0.84) | <0.001 | 1.84 (0.35) | 6.51 (1.13) | <0.0001 | 2.29 (0.43) | 11.09 (1.26) | <0.001 |

SDS=standard deviation score; BMI=body mass index.

Continuous variables are presented as the mean (standard error) and categorical data as the percentage (standard error).

Supplementary Table 2. Comparison of subjects according to suicidal ideation

|  | Total | |  | Male | |  | Female | |  |
| --- | --- | --- | --- | --- | --- | --- | --- | --- | --- |
|  | Yes | No | *p* | Yes | No | *p* | Yes | No | *p* |
| Age, y | 15.21 (0.09) | 15.10 (0.03) | 0.223 | 15.26 (0.14) | 15.10 (0.04) | 0.254 | 15.18 (0.11) | 15.10 (0.05) | 0.505 |
| Height SDS | 0.11 (0.05) | 0.226 (0.02) | 0.025 | 0.17 (0.075) | 0.24 (0.03) | 0.393 | 0.07 (0.07) | 0.22 (0.02) | 0.033 |
| Weight SDS | 0.11 (0.06) | 0.073 (0.02) | 0.495 | 0.13 (0.11) | 0.05 (0.03) | 0.516 | 0.11 (0.07) | 0.10(0.03) | 0.857 |
| BMI SDS | 0.08 (0.07) | -0.056 (0.02) | 0.061 | 0.05 (0.13) | -0.10 (0.03) | 0.274 | 0.09 (0.08) | -0.01 (0.03) | 0.200 |
| Rural area | 13.39 (2.06) | 15.27 (1.09) | 0.370 | 16.37 (3.23) | 14.71 (1.11) | 0.592 | 11.61 (2.24) | 15.95 (1.34) | 0.092 |
| Income below median | 39.22 (2.57) | 39.17 (1.00) | 0.982 | 31.87 (4.08) | 39.35 (1.19) | 0.086 | 43.67 (3.13) | 38.94 (1.32) | 0.138 |
| Mother graduated university | 31.36 (2.50) | 34.87 (1.02) | 0.171 | 36.91 (4.43) | 32.80 (1.20) | 0.350 | 28.05 (2.83) | 37.39 (1.37) | 0.003 |
| Father graduated university | 44.03 (3.20) | 46.30 (1.28) | 0.486 | 46.13 (5.11) | 46.10 (1.53) | 0.100 | 42.58 (3.96) | 46.54 (1.65) | 0.351 |
| Drinking | 35.92 (2.34) | 25.72 (0.75) | <0.001 | 36.92 (3.89) | 28.28 (1.00) | 0.022 | 35.33 (2.91) | 22.59 (1.06) | <0.001 |
| Drinking mother | 72.71 (2.51) | 75.91 (0.81) | 0.199 | 73.27 (3.80) | 75.58 (1.06) | 0.543 | 72.38 (3.00) | 76.30 (1.09) | 0.197 |
| Drinking father | 86.05 (2.57) | 88.13 (0.74) | 0.400 | 84.38 (3.96) | 87.80 (0.93) | 0.351 | 87.19 (2.78) | 88.51 (1.02) | 0.646 |
| Smoking | 11.53 (1.66) | 7.58 (0.47) | 0.009 | 17.48 (3.30) | 11.35 (0.76) | 0.035 | 8.01 (1.84) | 2.99 (0.42) | <0.001 |
| Smoking mother | 14.60 (1.914) | 9.00 (0.565) | <0.001 | 14.21 (3.07) | 9.26 (0.68) | 0.062 | 14.83 (2.47) | 8.68 (0.76) | 0.005 |
| Smoking father | 79.30 (2.733) | 84.49 (0.805) | 0.041 | 76.05 (4.65) | 86.01 (0.95) | 0.012 | 81.51 (2.92) | 82.70 (1.14) | 0.694 |
| Sleep duration, h/day | 6.84 (0.07) | 7.31 (0.02) | <0.001 | 6.81 (0.126) | 7.38 (0.031) | <0.001 | 6.86 (0.09) | 7.23 (0.04) | <0.001 |
| High stress | 65.40 (2.31) | 23.06 (0.68) | <0.001 | 63.04 (3.85) | 20.56 (0.87) | <0.001 | 66.80 (2.80) | 26.14 (1.04) | <0.001 |
| Mother with high stress | 36.71 (2.58) | 27.52 (0.92) | <0.001 | 33.55 (4.30) | 27.37 (1.15) | 0.143 | 38.59 (3.23) | 27.70 (1.20) | <0.001 |
| Father with high stress | 30.93 (2.87) | 28.17 (1.02) | 0.344 | 28.17 (4.34) | 28.27 (1.26) | 0.981 | 32.82 (3.73) | 28.05 (1.32) | 0.207 |
| Stress |  |  | <0.001 |  |  | 0.508 |  |  | <0.001 |
| Very low | 2.48 (0.71) | 17.24 (0.59) |  | 8.18 (2.65) | 9.97 (0.75) |  | 2.14 (0.82) | 15.46 (0.82) |  |
| Low | 32.12 (2.28) | 59.69 (0.77) |  | 58.27 (4.56) | 62.66 (1.22) |  | 31.06 (2.76) | 58.40 (1.13) |  |
| High | 50.32 (2.40) | 20.46 (0.66) |  | 28.38 (4.07) | 23.75 (1.120) |  | 49.57 (2.89) | 23.11 (1.00) |  |
| Very high | 15.07 (1.742) | 2.61 (0.253) |  | 5.17 (2.10) | 3.62 (0.44) |  | 17.23 (2.26) | 3.03 (0.41) |  |
| Stress of mother |  |  | 0.002 |  |  | 0.508 |  |  | 0.003 |
| Very low | 6.48 (1.28) | 9.44 (0.57) |  | 8.18 (2.65) | 9.97 (0.75) |  | 5.48 (1.30) | 8.81 (0.76) |  |
| Low | 56.80 (2.72) | 63.04 (0.96) |  | 58.27 (4.56) | 62.66 (1.22) |  | 55.94 (3.24) | 63.49 (1.27) |  |
| High | 31.10 (2.50) | 23.79 (0.89) |  | 28.38 (4.07) | 23.75 (1.12) |  | 32.70 (3.20) | 23.83 (1.14) |  |
| Very high | 5.62 (1.29) | 3.73 (0.37) |  | 5.17 (2.10) | 3.62 (0.44) |  | 5.88 (1.50) | 3.87 (0.53) |  |
| Stress of father |  |  | 0.575 |  |  | 0.542 |  |  | 0.591 |
| Very low | 9.26 (1.77) | 11.25 (0.70) |  | 8.27 (2.69) | 12.51 (0.95) |  | 9.93 (2.35) | 9.76 (0.84) |  |
| Low | 59.81 (3.10) | 60.58 (1.10) |  | 63.56 (4.80) | 59.22 (1.39) |  | 57.25 (3.87) | 62.19 (1.44) |  |
| High | 25.73 (2.68) | 24.20 (0.97) |  | 22.69 (4.00) | 24.04 (1.19) |  | 27.81 (3.52) | 24.40 (1.28) |  |
| Very high | 5.20 (1.44) | 3.96 (0.42) |  | 5.47 (2.29) | 4.23 (0.57) |  | 5.02 (1.83) | 3.65 (0.53) |  |
| Depressed mood | 38.10 (2.45) | 6.66 (0.40) | <0.001 | 36.76 (3.99) | 5.62 (0.50) | <0.001 | 38.90 (2.87) | 7.93 (0.65) | <0.001 |
| Mother with depressed mood | 18.67 (2.23) | 13.05 (0.78) | 0.007 | 15.95 (3.37) | 13.96 (1.03) | 0.550 | 20.20 (2.76) | 11.93 (0.96) | 0.001 |
| Father with depressed mood | 13.73 (2.57) | 7.66 (0.70) | 0.005 | 15.25 (4.13) | 7.67 (0.85) | 0.022 | 12.75 (2.81) | 7.65 (0.95) | 0.044 |
| Mother with suicidal ideation | 19.85 (2.41) | 10.70 (0.73) | <0.001 | 19.61 (4.02) | 10.88 (0.86) | 0.010 | 19.99 (2.720) | 10.48 (1.04) | <0.001 |
| Father with suicidal ideation | 13.95 (2.55) | 6.28 (0.59) | <0.001 | 12.84 (3.77) | 6.63 (0.80) | 0.039 | 14.68 (2.90) | 5.85 (0.75) | <0.001 |
| Suicidal plan | 27.29 (4.56) | 0.00 (0.000) | <0.001 | 21.86 (6.83) | 0.00 (0.00) | <0.001 | 31.08 (6.279) | 0.00 (0.000) | <0.001 |
| Suicidal attempt | 11.75 (1.59) | 0.01 (0.007) | <0.001 | 11.96 (2.81) | 0.00 (0.00) | <0.001 | 11.62 (1.91) | 0.01 (0.01) | <0.001 |
| Psychological consultation | 14.80 (1.89) | 2.78 (0.28) | <0.001 | 12.82 (3.01) | 2.25 (0.34) | <0.001 | 15.92 (2.39) | 3.43 (0.45) | <0.001 |

SDS=standard deviation score; BMI=body mass index.

Continuous variables are presented as the mean (standard error) and categorical data as the percentage (standard error).

Supplementary table 3. Comparison of the subjects according to mother's depressed mood

|  | Total | |  | Male | |  | Female | |  |
| --- | --- | --- | --- | --- | --- | --- | --- | --- | --- |
|  | Yes | No | *p* | Yes | No | *p* | Yes | No | *p* |
| Drinking | 33.75 (2.70) | 24.49 (0.94) | <0.001 | 34.70 (3.48) | 27.35 (1.29) | 0.035 | 32.59 (3.67) | 21.26 (1.27) | 0.001 |
| Smoking | 12.67 (1.81) | 6.63 (0.53) | <0.001 | 17.59 (2.85) | 10.00 (0.87) | 0.002 | 6.71 (1.87) | 2.84 (0.48) | 0.007 |
| High stress | 32.21 (2.39) | 26.09 (0.90) | 0.012 | 26.76 (3.07) | 22.50 (1.15) | 0.175 | 38.91 (3.43) | 30.14 (1.27) | 0.014 |
| Stress |  |  | 0.007 |  |  | 0.560 |  |  | <0.001 |
| Very low | 13.53 (1.74) | 16.40 (0.70) |  | 16.79 (2.64) | 18.44 (1.02) |  | 9.53 (2.19) | 14.10 (0.96) |  |
| Low | 54.26 (2.65) | 57.51 (0.97) |  | 56.45 (3.61) | 59.06 (1.31) |  | 51.56 (3.64) | 55.76 (1.39) |  |
| High | 25.43 (2.21) | 22.49 (0.88) |  | 23.77 (3.00) | 19.63 (1.10) |  | 27.46 (2.95) | 25.71 (1.22) |  |
| Very high | 6.78 (1.32) | 3.60 (0.37) |  | 2.98 (1.10) | 2.87 (0.46) |  | 11.45 (2.52) | 4.43 (0.56) |  |
| Depressed mood | 15.95 (1.81) | 8.47 (0.60) | <0.001 | 11.50 (2.13) | 7.32 (0.76) | 0.036 | 21.42 (2.92) | 9.78 (0.89) | <0.001 |
| Suicidal ideation | 14.34 (1.72) | 9.87 (0.59) | 0.006 | 7.98 (1.71) | 6.91 (0.71) | 0.550 | 22.16 (2.99) | 13.21 (0.91) | 0.001 |
| Suicidal plan | 2.61 (1.55) | 0.46 (0.22) | 0.011 | 0.00 (0.00) | 0.00 (0.00) | >0.999 | 5.21 (3.08) | 0.97 (0.46) | 0.015 |
| Suicidal attempt | 3.47 (1.27) | 2.03 (0.42) | 0.204 | 0.00 (0.00) | 1.57 (0.57) | <0.001 | 6.05 (2.22) | 2.49 (0.64) | 0.045 |
| Psychological consultation | 7.14 (1.38) | 2.87 (0.34) | <0.001 | 4.52 (1.43) | 2.24 (0.40) | 0.051 | 10.31 (2.39) | 3.57 (0.56) | <0.001 |

Values are presented as the percentage (standard error).

|  | Total | |  | Male | |  | Female | |  |
| --- | --- | --- | --- | --- | --- | --- | --- | --- | --- |
|  | Yes | No | *p* | Yes | No | *p* | Yes | No | *p* |
| Drinking | 31.69 (2.62) | 24.98 (0.94) | 0.009 | 32.56 (3.46) | 27.86 (1.31) | 0.184 | 30.72 (4.11) | 21.68 (1.27) | 0.024 |
| Smoking | 11.62 (1.77) | 6.90 (0.54) | 0.002 | 16.35 (2.73) | 10.37 (0.90) | 0.013 | 6.40 (2.03) | 2.93 (0.48) | 0.027 |
| High stress | 31.63 (2.40) | 26.26 (0.92) | 0.032 | 27.92 (3.18) | 22.41 (1.17) | 0.093 | 35.76 (3.37) | 30.69 (1.28) | 0.156 |
| Stress |  |  | 0.010 |  |  | 0.299 |  |  | 0.023 |
| Very low | 12.93 (1.77) | 16.44 (0.72) |  | 15.89 (2.68) | 18.54 (1.05) |  | 9.65 (2.26) | 14.02 (0.95) |  |
| Low | 55.44 (2.73) | 57.30 (0.99) |  | 56.20 (3.82) | 59.05 (1.33) |  | 54.60 (3.61) | 55.29 (1.39) |  |
| High | 24.65 (2.14) | 22.62 (0.90) |  | 23.40 (2.97) | 19.74 (1.14) |  | 26.04 (2.88) | 25.92 (1.23) |  |
| Very high | 6.98 (1.44) | 3.65 (0.37) |  | 4.52 (1.63) | 2.67 (0.43) |  | 9.72 (2.36) | 4.76 (0.60) |  |
| Depressed mood | 14.13 (1.95) | 8.88 (0.58) | 0.003 | 13.12 (2.63) | 7.23 (0.72) | 0.008 | 15.26 (2.90) | 10.77 (0.91) | 0.103 |
| Suicidal ideation | 17.84 (2.24) | 9.51 (0.56) | <0.001 | 12.04 (2.61) | 6.41 (0.66) | 0.010 | 24.27 (3.34) | 13.06 (0.91) | <0.001 |
| Suicidal plan | 5.05 (3.64) | 0.51 (0.22) | 0.001 | 0.00 (0.00) | 0.00 (0.00) | >0.999 | 10.55 (7.67) | 1.07 (0.46) | 0.001 |
| Suicidal attempt | 3.26 (1.45) | 2.11 (0.41) | 0.371 | 0.00 (0.00) | 1.51 (0.55) | <0.001 | 5.53 (2.49) | 2.68 (0.64) | 0.153 |
| Psychological consultation | 4.58 (1.26) | 3.31 (0.36) | 0.275 | 4.35 (1.70) | 2.33 (0.39) | 0.141 | 4.84 (1.65) | 4.44 (0.62) | 0.817 |

Supplementary table 4. Comparison of the subjects according to mother's suicidal ideation

Values are presented as the percentage (standard error).

Supplementary table 5. Univariable logistic regression for stress

|  | Total | | Male | | Female | |
| --- | --- | --- | --- | --- | --- | --- |
|  | OR (95% CI) | *p* | OR (95% CI) | *p* | OR (95% CI) | *p* |
| Age, y | 1.076 (1.041-1.112) | <0.001 | 1.033 (0.986-1.082) | 0.168 | 1.112 (1.069-1.173) | <0.001 |
| Area |  |  |  |  |  |  |
| Urban | ref |  | ref |  | ref |  |
| Rural | 0.872 (0.712-1.066) | 0.182 | 0.901 (0.701-1.160) | 0.419 | 0.836 (0.643-1.086) | 0.180 |
| Income |  |  |  |  |  |  |
| Below median | ref |  | ref |  | ref |  |
| Above median | 0.847 (0.735-0.976) | 0.022 | 0.918 (0.753-1.119) | 0.396 | 0.786 (0.649-0.952) | 0.014 |
| Mother education |  |  |  |  |  |  |
| -High school | ref |  | ref |  | ref |  |
| University- | 1.073 (0.921-1.252) | 0.367 | 1.158 (0.933-1.437) | 0.182 | 0.975 (0.791-1.201) | 0.813 |
| Father education |  |  |  |  |  |  |
| -High school | ref |  | ref |  | ref |  |
| University- | 0.960 (0.805-1.146) | 0.652 | 1.020 (0.797-1.306) | 0.876 | 0.906 (0.719-1.142) | 0.403 |
| Drinking | 1.273 (1.104-1.467) | <0.001 | 1.110 (0.905-1.362) | 0.316 | 1.553 (1.261-1.913) | <0.001 |
| Drinking mother | 1.078 (0.914-1.271) | 0.376 | 1.258 (0.903-1.754) | 0.175 |  |  |
| Drinking father | 1.164 (0.904-1.500) | 0.240 | 1.303 (0.827-2.052) | 0.254 |  |  |
| Smoking | 1.553 (1.214-1.998) | <0.001 | 1.654 (1.226-2.231) | 0.001 | 2.160 (1.326-3.518) | 0.002 |
| Smoking mother | 1.624 (1.284-2.054) | <0.001 | 1.657 (1.211-2.267) | 0.002 | 1.609 (1.145-2.262) | 0.006 |
| Smoking father | 1.089 (0.860-1.381) | 0.479 | 0.975 (0.693-1.372) | 0.885 | 1.242 (0.909-1.697) | 0.175 |
| Sleep duration, h/day | 0.826 (0.785-0.868) | <0.001 | 0.851 (0.788-0.918) | <0.001 | 0.815 (0.764-0.870) | <0.001 |
| Mother with high stress | 1.386 (1.183-1.623) | <0.001 | 1.429 (1.141-1.788) | 0.002 | 1.338 (1.083-1.653) | 0.007 |
| Father with high stress | 1.168 (0.974-1.400) | 0.0941 | 0.999 (0.768-1.301) | 0.996 | 1.348 (1.059-1.717) | 0.015 |
| Stress of mother |  |  |  |  |  |  |
| Very low | ref |  | ref |  | ref |  |
| Low | 1.266 (0.938-1.710) | 0.123 | 1.084 (0.717-1.638) | 0.039 | 1.459 (1.002-2.126) | 0.409 |
| High | 1.639 (1.192-2.254) | 0.002 | 1.471 (0.947-2.286) | 0.349 | 1.802 (1.202-2.702) | 0.213 |
| Very high | 2.163 (1.354-3.454) | 0.001 | 1.954 (1.027-3.719) | 0.054 | 2.346 (1.265-4.351) | 0.045 |
| Stress of father |  |  |  |  |  |  |
| Very low | ref |  | ref |  | ref |  |
| Low | 0.990 (0.755-1.300) | 0.120 | 0.896 (0.617-1.301) | 0.347 | 1.075 (0.729-1.584) | 0.186 |
| High | 1.126 (0.828-1.531) | 0.876 | 0.858 (0.564-1.304) | 0.267 | 1.424 (0.936-2.167) | 0.225 |
| Very high | 1.363 (0.854-2.176) | 0.186 | 1.247 (0.643-2.418) | 0.302 | 1.507 (0.798-2.848) | 0.340 |
| Depressed mood | 6.717 (5.432-8.305) | <0.001 | 6.353 (4.611-8.754) | <0.001 | 6.685 (5.001-8.937) | <0.001 |
| Mother with depressed mood | 1.346 (1.066-1.699) | 0.012 | 1.258 (0.903-1.754) | 0.175 | 1.476 (1.081-2.015) | 0.014 |
| Father with depressed mood | 1.217 (0.878-1.686) | 0.238 | 1.303 (0.827-2.052) | 0.254 | 1.136 (0.724-1.784) | 0.579 |
| Suicidal ideation | 6.304 (5.093-7.802) | <0.001 | 6.588 (4.674-9.284) | <0.001 | 5.685 (4.342-7.444) | <0.001 |
| Mother with suicidal ideation | 1.299 (1.022-1.651) | 0.032 | 1.341 (0.951-1.891) | 0.094 | 1.257 (0.917-1.725) | 0.156 |
| Father with suicidal ideation | 1.358 (0.975-1.890) | 0.070 | 1.290 (0.780-2.130) | 0.327 | 1.444 (0.929-2.245) | 0.103 |

OR=odds ratio; CI=confidence interval

Supplementary table 6. Multivariable logistic regression with stepwise selection for stress

|  | Total | | Male | | Female | |
| --- | --- | --- | --- | --- | --- | --- |
|  | OR (95% CI) | *p* | OR (95% CI) | *p* | OR (95% CI) | *p* |
| Income |  |  |  |  |  |  |
| Below median | ref |  | ref |  | ref |  |
| Above median | 0.819 (0.690-0.971) | 0.022 |  |  |  |  |
| Smoking |  |  | 1.443 (1.000-2.083) | 0.050 | 1.973 (1.022-3.807) | 0.043 |
| Smoking mother | 1.385 (1.069-1.794) | 0.014 | 1.492 (1.081-2.060) | 0.015 |  |  |
| Sleep duration, h/day | 0.844 (0.798-0.893) | <0.001 | 0.912 (0.838-0.991) | 0.030 | 0.879 (0.803-0.962) | 0.005 |
| Mother with high stress | 1.204 (1.013-1.432) | 0.036 | 1.276 (1.005-1.620) | 0.045 |  |  |
| Father with high stress |  |  |  |  | 1.337 (1.027-1.740) | 0.031 |
| Depressed mood | 4.160 (3.216-5.382) | <0.001 | 3.692 (2.536-5.374) | <0.001 | 4.592 (3.029-6.963) | <0.001 |
| Suicidal ideation | 3.909 (3.050-5.011) | <0.001 | 3.695 (2.477-5.510) | <0.001 | 4.144 (2.837-6.051) | <0.001 |

OR=odds ratio; CI=confidence interval

Supplementary table 7. Univariable logistic regression for suicidal ideation

|  | Total | | Male | | Female | |
| --- | --- | --- | --- | --- | --- | --- |
|  | OR (95% CI) | *p* | OR (95% CI) | *p* | OR (95% CI) | *p* |
| Age, y | 1.030 (0.982-1.079) | 0.225 | 1.044 (0.969-1.126) | 0.257 | 1.021 (0.960-1.087) | 0.506 |
| Area |  |  |  |  |  |  |
| Urban | ref |  | ref |  | ref |  |
| Rural | 0.858 (0.613-1.200) | 0.371 | 1.134 (0.715-1.799) | 0.592 | 0.693 (0.450-1.066) | 0.095 |
| Income |  |  |  |  |  |  |
| Below median | ref |  | ref |  | ref |  |
| Above median | 0.998 (0.805-1.235) | 0.982 | 1.387 (0.954-2.017) | 0.087 | 0.823 (0.636-1.065) | 0.138 |
| Mother education |  |  |  |  |  |  |
| -High school | ref |  | ref |  | ref |  |
| University- | 0.853 (0.679-1.072) | 0.172 | 1.199 (0.820-1.752) | 0.350 | 0.653 (0.490-0.869) | 0.004 |
| Father graduated university |  |  |  |  |  |  |
| -High school | ref |  | ref |  | ref |  |
| University- | 0.912 (0.704-1.182) | 0.487 | 1.001 (0.663-1.512) | 0.996 | 0.852 (0.608-1.194) | 0.352 |
| Drinking | 1.619 (1.313-1.995) | <0.001 | 1.485 (1.058-2.084) | 0.022 | 1.872 (1.428-2.453) | <0.001 |
| Drinking mother | 0.846 (0.655-1.092) | 0.199 | 0.886 (0.599-1.310) | 0.543 | 0.814 (0.595-1.113) | 0.198 |
| Drinking father | 0.831 (0.539-1.281) | 0.401 | 0.750 (0.401-1.374) | 0.352 | 0.883 (0.519-1.502) | 0.646 |
| Smoking | 1.589 (1.121-2.253) | 0.009 | 1.655 (1.032-2.653) | 0.036 | 2.826 (1.603-4.981) | <0.001 |
| Smoking mother | 1.729 (1.254-2.384) | <0.001 | 1.624 (0.971-2.715) | 0.065 | 1.831 (1.200-2.793) | 0.005 |
| Smoking father | 0.703 (0.501-0.987) | 0.042 | 0.517 (0.306-0.872) | 0.013 | 0.922 (0.616-1.381) | 0.694 |
| Sleep duration, h/day | 0.793 (0.736-0.855) | <0.001 | 0.743 (0.649-0.851) | <0.001 | 0.843 (0.769-0.923) | <0.001 |
| High stress | 6.304 (5.093-7.803) | <0.001 | 6.590 (4.676-9.288) | <0.001 | 5.685 (4.342-7.444) | <0.001 |
| Stress |  |  |  |  |  |  |
| Very low | ref |  | ref |  | ref |  |
| Low | 3.742 (2.059-6.801) | <0.001 | 3.416 (1.383-8.441) | <0.001 | 3.845 (1.739-8.500) | <0.001 |
| High | 17.104 (9.524-30.717) | <0.001 | 17.257 (7.016-42.447) | <0.001 | 15.501 (7.073-33.968) | <0.001 |
| Very high | 40.220 (20.924-77.310) | <0.001 | 30.952 (10.8946-87.938) | <0.001 | 41.154 (17.461-96.998) | <0.001 |
| Mother with high stress | 1.528 (1.219-1.915) | <0.001 | 1.340 (0.906-1.983) | 0.143 | 1.640 (1.232-2.182) | <0.001 |
| Father with high stress | 1.142 (0.868-1.503) | 0.344 | 0.995 (0.643-1.538) | 0.981 | 1.254 (0.883-1.780) | 0.207 |
| Stress of mother |  |  |  |  |  |  |
| Very low | ref |  | ref |  | ref |  |
| Low | 1.313 (0.851-2.025) | 0.128 | 1.133 (0.551-2.332) | 0.417 | 1.417 (0.833-2.412) | 0.180 |
| High | 1.904 (1.225-2.961) | 0.050 | 1.456 (0.684-3.100) | 0.560 | 2.207 (1.248-3.905) | 0.048 |
| Very high | 2.195 (1.142-4.219) | 0.074 | 1.743 (0.576-5.278) | 0.398 | 2.448 (1.136-5.275) | 0.097 |
| Stress of father |  |  |  |  |  |  |
| Very low | ref |  | ref |  | ref |  |
| Low | 1.200 (0.772-1.864) | 0.704 | 1.623 (0.786-3.354) | 0.554 | 0.905 (0.523-1.566) | 0.229 |
| High | 1.292 (0.804-2.074) | 0.823 | 1.428 (0.658-3.098) | 0.918 | 1.120 (0.606-2.071) | 0.843 |
| Very high | 1.595 (0.787-3.233) | 0.298 | 1.956 (0.642-5.957) | 0.409 | 1.353 (0.532-3.440) | 0.474 |
| Depressed mood | 8.626 (6.804-10.937) | <0.001 | 9.755 (6.690-14.224) | <0.001 | 7.388 (5.552-9.830) | <0.001 |
| Mother with depressed mood | 1.530 (1.126-2.079) | 0.007 | 1.169 (0.700-1.952) | 0.550 | 1.870 (1.281-2.729) | 0.001 |
| Father with depressed mood | 1.918 (1.209-3.043) | 0.006 | 2.165 (1.101-4.257) | 0.025 | 1.763 (1.007-3.089) | 0.047 |
| Mother with suicidal ideation | 2.067 (1.487-2.872) | <0.001 | 1.998 (1.170-3.412) | 0.011 | 2.134 (1.436-3.170) | <0.001 |
| Father with suicidal ideation | 2.422 (1.554-3.774) | <0.001 | 2.076 (1.023-4.214) | 0.043 | 2.770 (1.668-4.5980) | <0.001 |

OR=odds ratio; CI=confidence interval

Supplementary table 8. Multivariable logistic regression with stepwise selection for suicidal ideation

|  | Total | | Male | | Female | |
| --- | --- | --- | --- | --- | --- | --- |
|  | OR (95% CI) | *p* | OR (95% CI) | *p* | OR (95% CI) | *p* |
| Smoking father | 0.627 (0.416-0.945) | 0.026 | 0.502 (0.283-0.888) | 0.018 |  |  |
| Sleep duration, h/day | 0.829 (0.737-0.933) | 0.002 | 0.725 (0.614-0.856) | <0.001 |  |  |
| High stress |  |  | 4.311 (2.695-6.895) | <0.001 |  |  |
| Stress |  |  |  |  |  |  |
| Very low | ref |  | ref |  | ref |  |
| Low | 10.387 (3.968-27.189) | <0.001 |  |  | 4.167 (1.706-10.177) | 0.002 |
| High | 28.918 (10.907-76.670) | <0.001 |  |  | 12.295 (4.950-30.537) | <0.001 |
| Very high | 49.645 (17.057-144.494) | <0.001 |  |  | 20.402 (6.939-59.987) | <0.001 |
| Depressed mood | 5.990 (4.183-8.578) | <0.001 | 7.116 (4.150-12.202) | <0.001 | 5.077 (3.197-8.063) | <0.001 |
| Mother with suicidal ideation | 1.683 (1.025-2.761) | 0.040 |  |  | 1.683 (0.954-2.968) | 0.072 |
| Father with suicidal ideation | 2.581 (1.469-4.535) | 0.001 |  |  | 2.702 (1.497-4.877) | 0.001 |

OR=odds ratio; CI=confidence interval
